# Smoking and the risk of acute myeloid leukaemia in cytogenetic subgroups

**DOI:** 10.1038/sj.bjc.6600010

**Published:** 2002-01-07

**Authors:** A V Moorman, E Roman, R A Cartwright, G J Morgan

**Affiliations:** Leukaemia Research Fund Centre for Clinical Epidemiology, University of Leeds, 30 Hyde Terrace, Leeds LS2 9LN, UK; Department of Haematology, Leeds General Infirmary, Leeds, UK

**Keywords:** cytogenetics, chromosomal abnormalities, tobacco smoke, acute myeloid leukaemia, aetiology

## Abstract

Cytogenetically-defined subgroups of acute myeloid leukaemia have distinct biologies, clinical features and outcomes. Evidence from therapy-related leukaemia suggests that chromosomal abnormalities are also markers of exposure. Our results suggest that the smoking-associated risk for acute myeloid leukaemia is restricted to the t(8;21)(q22;q22) subgroup. This supports the hypothesis that distinct cytogenetic subgroups of acute myeloid leukaemia have separate aetiologies.

*British Journal of Cancer* (2002) **86**, 60–62. DOI: 10.1038/sj/bjc/6600010 www.bjcancer.com

© 2002 The Cancer Research Campaign

## 

In spite of extensive research into the aetiology of acute myeloid leukaemia (AML) the cause of the majority of cases remains unknown. Data from therapy-related leukaemia suggest that different carcinogens may induce leukaemias, via separate mechanisms, with distinct chromosomal abnormalities. For example, AML which develops after treatment with drugs targeting DNA topoisomerase II is characterized by the presence of balanced translocations, especially those involving the *MLL* gene located on chromosome 11 at q23 (Andersen *et al*, 1998). In contrast, unbalanced aberrations (e.g. −5, del(5q), −7 and del(7q)) predominate in AML induced by alkylating agents (Pedersen-Bjergaard *et al*, 1993). Therefore, using cytogenetics to define subtypes of AML may help to identify risk factors more readily.

Although leukaemia is not considered one of the major smoking-related cancers, evidence from a number of cohort and case–control studies does indicate a weak association. Cohorts of British doctors and US veterans have both shown small but significant increases in the number of ever smokers developing leukaemia when compared with life-long non-smokers (Doll *et al*, 1994; McLaughlin *et al*, 1995). Similar results have also been obtained from a number of case–controls studies, including Brownson *et al* (1991), Pasqualetti *et al* (1997) and Kane *et al* (1999). Overall, the increased risk appears to be confined to the ‘acute’ and ‘myeloid’ forms of the disease, rather than the ‘lymphoid’ or ‘chronic’ forms. The most recent case–control study reported an odds ratio (OR) of 1.2 (95% confidence intervals (CI) (1.0, 1.4)) for the risk of developing AML associated with ever smoking (Kane *et al*, 1999). The effect was strongest for current smoking (OR=1.4, 95% CI (1.1, 1.8)) and was absent among ex-smokers (OR=0.9, 95% CI (0.7, 1.2)). Furthermore, some studies have reported that the risk may be confined to certain cytogenetic subgroups (Crane *et al*, 1989, 1996; Sandler *et al*, 1993). However, the results have been inconsistent, possibly due to the small number of cases in each cytogenetic subgroup. In this report the analysis presented by Kane *et al* (1999) has been extended to estimate the smoking-associated risk of AML in the five most frequent cytogenetic subgroups.

## PATIENTS AND METHODS

This study was based on subjects from a case–control study of acute leukaemia which has been described in detail elsewhere (Kane *et al*, 1999). Briefly, the study ascertained adults (16–69 years-old) diagnosed with acute leukaemia over a 5-year period in parts of the north and southwest of England. Controls were randomly selected from persons registered with the same local physician as the case. Smoking histories were collected during a face-to-face interview where smoking was defined as at least one cigarette per day for a minimum of 6 months. Each subject was classified as a never, current or past smoker, assuming a 2-year lag period prior to diagnosis.

The current analysis has been restricted to patients with a pathologically confirmed diagnosis of *de novo* AML. Diagnostic cytogenetic data for the cases were collected from regional laboratories (Moorman *et al*, 2001). Each case was classified, according to the clonal aberrations observed in the main leukaemic clone, into one of five cytogenetic groups: t(15;17)(q22;q12), t(8;21) (q22;q22), inv(16)(p13q22), del(5q)/−5/del(7q)/−7 and +8. Cases harbouring two or more of these abnormalities were placed into the first group in the list. Other abnormalities occurred too infrequently to be considered separately and were therefore grouped together; as were cases where no abnormality was detected.

Odds ratios (OR) and 95% confidence intervals (CI) were estimated using individual logistic regression models, comparing cases in each cytogenetic subgroup to all controls; adjusting for age, sex, region and deprivation. Three comparisons were made: (1) ever versus never smoking; (2) current versus never smoking; and (3) past versus never smoking. All analyses were performed using Intercooled Stata 6.0 for Windows (Stata Corporation, 1999).

## RESULTS AND DISCUSSION

Among 600 cases cytogenetics was successful for 472 (79%) cases, while 24 (4%) cases failed cytogenetics and 104 (17%) cases were not tested. Overall, cases had a higher percentage of smokers, both ever and current, compared to the controls but fewer ex-smokers (Table 1

**Table 1:**
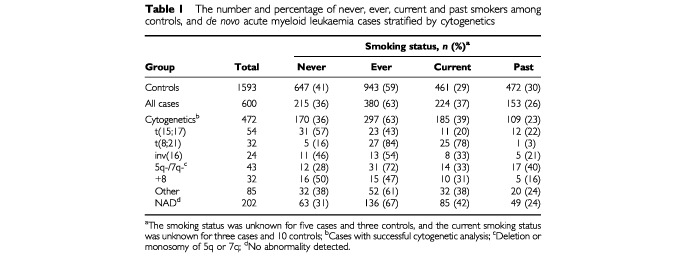
The number and percentage of never, ever, current and past smokers among controls, and *de novo* acute myeloid leukaemia cases stratified by cytogenetics

). A raised odds ratio was observed for ever smoking (OR=1.19) but the risk was confined to current smokers (OR=1.42) with no effect being seen among the ex-smokers (OR=0.94) (Table 2

**Table 2:**
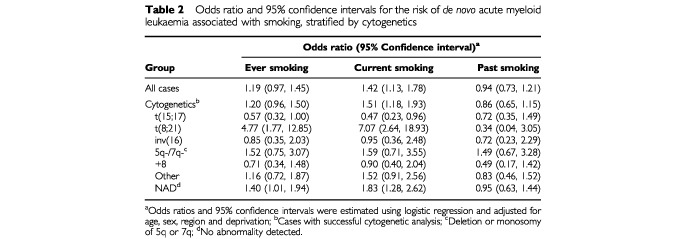
Odds ratio and 95% confidence intervals for the risk of *de novo* acute myeloid leukaemia associated with smoking, stratified by cytogenetics

). There was some indication of variation between the cytogenetic subgroups (Tables 1 and 2). Among 32 cases with t(8;21), 27 (84%) had smoked at some point during their lives and 25 (78%) were known to be current smokers. Within the t(8;21) subgroup, ever and current smoking were associated with a five- and seven-fold increased risk of AML (OR=4.77 and 7.07) but no risk was seen among ex-smokers (OR=0.34). In contrast, only 23 out of 54 (43%) t(15;17) cases were ever smokers of which half had given up the habit. Hence within this subgroup the ORs for ever, current and past smoking were all reduced (OR=0.57, 0.47 and 0.72). The estimates of risk in the other cytogenetic subgroups were either very similar to the risk observed in the whole group or to unity (Table 2).

These findings are supported by a US-based study who reported that ever smoking increased the risk of t(8;21) positive AML (OR=1.71, 95% CI (0.60, 5.13)) while also observing a reduced OR for the t(15;17) group (OR=0.42, 95% CI (0.17, 1.01)) (Sandler *et al*, 1993). However, neither result reached statistical significance, probably due to the number of cases in each group: 26 and 19 cases respectively. Sandler *et al* (1993) also reported an increased risk for the –7/del(7q) group (OR=7.91, 95% CI (1.04, 166)) which was not observed in this study (data not shown) and is in contrast to the reduced OR (OR=0.2, 95% CI (0.1, 0.9)) observed by Crane *et al* (1989). Direct comparisons with the two studies by Crane *et al* (1989), (1996) are difficult because their reference group consisted of cases where no abnormality had been detected, as opposed to disease-free controls. However, their results also hinted at an association between ever smoking and t(8;21) positive AML (OR=2.3, 95% CI (0.8, 6.7) (Crane *et al*, 1989); OR=1.81, 95% CI (0.59, 6.51) (Crane *et al*, 1996)), even though both estimates were based on under 20 cases. Only one Crane study examined the t(15;17) subgroup and it showed a reduced OR (OR=0.4, 95% CI (0.1, 1.5)) (Crane *et al*, 1989). A recent Swedish study did not show any variation in the smoking-associated risk of AML among different cytogenetic subgroups, however it should be noted that the study was not large enough to examine the t(15;17) and t(8;21) subgroups separately (Bjork *et al*, 2001).

Although these results suggest the smoking-associated risk of *de novo* AML varies according to chromosomal abnormality, they should be interpreted with caution. Independent verification is needed because the number of cases in each subgroup was not large and it is possible that these are chance observations or confounded by other factors such as alcohol or diet for which we have no data. However, they do support a link between exposure and specific chromosomal aberrations. The mechanism by which smoking may cause AML and in particular t(8;21) positive AML is far from clear. However, tobacco smoke is the largest environmental source of benzene (Wallace, 1996) which is a well established risk factor for AML (Rinsky *et al*, 1987). Further indirect support comes from the observation that Chinese factory workers exposed to benzene showed a higher rate of translocations involving chromosomes 8 and 21 than controls; indeed one worker was shown to harbour the *ETO/AML1* fusion gene, the usual molecular consequence of t(8;21) (Smith *et al*, 1998). Future aetiological studies investigating AML may prove more fruitful if they include cytogenetic data and focus the analysis on subgroups of patients with identical or similar types of chromosomal abnormality.
